# Performance of Dried Blood Spots Compared with Serum Samples for Measuring Dengue Seroprevalence in a Cohort of Children in Cebu, Philippines

**DOI:** 10.4269/ajtmh.20-0937

**Published:** 2020-11-02

**Authors:** Jedas Veronica Daag, Michelle Ylade, Ramesh Jadi, Cameron Adams, Anna Maureen Cuachin, Riacarl Alpay, Emma Teresa Carmela Aportadera, In-Kyu Yoon, Aravinda M. de Silva, Anna Lena Lopez, Jacqueline Deen

**Affiliations:** 1 Institute of Child Health and Human Development, National Institutes of Health, University of the Philippines-Manila, Manila, Philippines;; 2 Department of Microbiology and Immunology, University of North Carolina School of Medicine, Chapel Hill, North Carolina;; 3 Coalition for Epidemic Preparedness Innovations, Washington, District of Columbia

## Abstract

Dengue seroprevalence data are useful for understanding epidemiologic trends and transmission dynamics, and for making decisions about implementation of dengue control programs. A logistical challenge to seroprevalence surveys is the collection and transport of serum samples. For conducting large and repeated dengue serosurveys, dried blood spots (DBS) would allow easier sample collection, shipment, transport, and storage than standard serum collection methods. Further evidence is needed to understand how well DBS performs compared with standard serum collection methods in laboratory assays. We evaluated the detection of anti-dengue antibodies by IgG indirect ELISA when using DBS compared with sera. Specimens were collected from healthy children in Cebu, Philippines, who would be 9–14 years of age at the time of a mass dengue vaccination program. Using an ELISA index value cutoff of 0.9, 1,285/1,488 (86.4%) of the DBS were seropositive and 203 (13.6%) were seronegative, compared with 1,292/1,488 (86.8%) seropositive and 196 (13.2%) seronegative serum samples. Compared with sera, the DBS method had a 98.3% sensitivity, 92.4% specificity, 98.9% positive predictive value, and 89.2% negative predictive value. Considering the advantages in terms of sample collection, shipment, and storage, DBS sampling may be appropriate for dengue population serosurveys.

## INTRODUCTION

Dengue fever is a mosquito-borne, acute febrile illness caused by four antigenically distinct dengue virus (DENV) serotypes, DENV-1 to 4. Estimates of symptomatic disease incidence provide important insights on the burden of dengue,^1^ but they are incomplete because of variable healthcare-seeking behavior and subclinical infections. Clinically apparent cases may be as low as 25% of total infections.^2,3^ Population-based serological surveys are an effective tool to determine the distribution and impact of dengue.^4^ Dengue seroprevalence data stratified by time, location, and age-group are useful for understanding seasonal and annual trends, spatial range, high-risk populations, and transmission dynamics. In addition, dengue seroprevalence data may also inform decisions on implementation or intensification of dengue control programs.^5^

The generally accepted reference standard for detecting anti-DENV antibodies from previous natural infection is neutralization testing of sera, but this method is time and labor intensive and only available in research settings.^6^ The less sensitive and specific but easier to perform indirect IgG ELISA has been recommended for population serosurveys, ideally with a subset validated by neutralization testing.^5^ We previously validated a dengue IgG indirect ELISA (PanBio, Brisbane, Australia) by neutralization testing using sera from healthy children in Cebu, Philippines.^7^ The ELISA with a 0.9 index value cutoff was 95.2% sensitive and 93.4% specific with a 6.6% false positivity and 4.8% false negativity compared with neutralization testing in the detection of dengue seropositivity.

Compared with sera, the use of dried blood spots (DBS) allows easier sample collection, shipment, transport, and storage, thus increasing accessibility of dengue serosurveys to rural areas and low-resource settings. Because blood volumes on DBS are small and the stability of the samples in a filter paper is uncertain, there is a need for rigorous assay validation compared with standard methods.^8^ We evaluated the performance of DBS as the index test compared with sera as the reference standard in the detection of previous DENV infection by dengue IgG ELISA.

## MATERIALS AND METHODS

The study was performed following the Standards for the Reporting of Diagnostic Accuracy Studies guidelines.^9^ The protocol was reviewed and approved by the University of the Philippines-Manila Research Ethics Board. A parent or legal guardian of the participants provided written informed consent. Verbal assent was obtained from the participants and documented. The study was conducted in accordance with the principles of the Declaration of Helsinki.

### Study participants and sample collection.

The Philippine government launched a dengue mass immunization in 2016 in high-risk regions using the first licensed dengue vaccine, CYD-TDV (Dengvaxia, Sanofi Pasteur, Lyon, France). We initiated a prospective cohort study just before the mass dengue vaccination in Cebu Province.^7^ We invited healthy children residing in Bogo and Balamban, semi-urban areas in Cebu, who would be 9–14 years of age at the time of the dengue mass vaccination to participate in the study. From each participant, we collected approximately 5 mL of blood in anticoagulant-free vacutainer tubes, which was processed and aliquoted. In the initial consecutive subset of participants, 60–70 µL of blood was blotted at the center of each collection card (Whatman 903 filter paper, Whatman plc, Maidstone, Kent, UK) immediately after the blood draw, using a disposable transfer pipette. The blood on the filter paper was allowed to air-dry for at least 4 hours on a rack. After completion of the drying process, the DBS were packed individually in single, gas-permeable zipper bags with three to five desiccant sachets and a humidity indicator, and kept at ambient temperature at the field sites. The frozen sera and DBS were shipped to the University of the Philippines Manila-NIH in dry ice then stored in −80°C before testing.

### Measurement of dengue IgG antibodies.

The serum samples were tested using dengue IgG indirect ELISA (PanBio) following the manufacturer’s instructions. In brief, 10 µL of thawed serum was mixed with 1000 µL of sample diluent (Tris-buffered saline). From the mixture, 100 µL was transferred to dengue antigen (serotypes 1, 2, 3, and 4) coated microwells and incubated for 30 minutes at 37°C. Each plate was washed six times to remove residual serum or any unbound materials. Horseradish peroxidase–conjugated goat antihuman IgG was added into each well and incubated again for another 30 minutes. Following a second washing, a colorless substrate system, tetramethylbenzidine/hydrogen peroxide (TMB Chromogen solution, Thermo Fisher Scientific, Waltham, MA), was pipetted into the wells. The substrate is hydrolyzed by the enzyme, and the chromogen changes to a blue color after a 10-minute incubation period. This reaction was stopped with the addition of an acid (1 M Phosphoric acid), changing the TMB to yellow. The absorbance of each well was read at 450 nm using a microplate reader. Color development is indicative of the presence of dengue IgG antibodies in the test samples. The ELISA index values were calculated by dividing the sample absorbance over the cutoff value (average absorbance of the triplicates of the calibrator multiplied by a batch calibration factor).

The DBS were tested using the same ELISA. The DBS were taken out of the freezer and allowed to equilibrate to room temperature for a minimum of 30 minutes before opening the storage bags. One circle of dried blood on a filter paper was punched into a microcentrifuge tube using a 6-mm device puncher (Whatman WB10040 Harris Unicore Punch). A single spot of dried blood yielded approximately three 6-mm filter paper circles. The amount of sera in each filter paper circle was estimated using the formula: 0.5 × (the volume of blood per spot divided by the number of filter paper circles per spot). Thus, each filter paper circle contained approximately 10–12 µL of sera (0.5 × [60–70 µL/3]), which is comparable to the required amount (10 µL) in serum ELISA testing. To prevent the potential risk of carryover contamination, we cleaned the punch device by wiping the tip with an ethanol-dipped paper towel between samples. One hundred fifty microliters of Panbio sample diluent (Tris-buffered saline) was added to the microcentrifuge tube and incubated at room temperature for about 2 hours with gentle rotation on a shaker. Tubes were then centrifuged at 10,000 g for 5 minutes to pellet any debris. Supernatant was transferred into a new tube. Fresh eluate was tested for the presence of dengue IgG antibodies, as described earlier.

The manufacturer’s recommended ELISA index cutoff values are as follows: an index value of < 0.9 is negative (no evidence of past dengue infection), 0.9–1.1 is equivocal, and > 1.1 is positive (presence of detectable IgG antibodies indicating a past or recent dengue infection). In our validation of the dengue IgG indirect ELISA for determining dengue serostatus, the receiver operating characteristic curve identified an optimal cutoff point of 0.859. Based on this, an IgG ELISA index value of < 0.9 is considered as dengue seronegative.^7^

The sera were batch tested from December 2017 to February 2018. The DBS were batch tested in July 2019. The laboratory technicians were blinded to the reference results using serum samples during the performance of the assay using DBS.

### Sample size and statistical analysis.

Using the sample size calculation by Buderer,^10^ assuming 80% seroprevalence,^1^ expected sensitivity and specificity of 95% and 98%, respectively, and desired precision of 0.02, we estimated that at least 946 samples would be needed. The primary endpoint of this study is the performance of DBS compared with sera in identifying dengue IgG ELISA binary results of seronegative (defined as an ELISA index value of less than 0.9) and seropositive (defined as an ELISA index value of 0.9 and greater).

First, we compared the demographic characteristics of participants enrolled in the study with those whose serum and DBS samples were tested by dengue IgG indirect ELISA using *t*-test for continuous variables and chi-squared test for categorical variables. Second, we examined the data by creating a scatterplot with a trend line and calculated the *R*-squared (*R*^2^) value. We created a Bland–Altman plot of the difference between the results from both methods against their mean.^11,12^ Plotting the difference between the results from the DBS and sera against their mean allows the identification of any systematic difference between the two measurements. If the points on the Bland–Altman plot are equally scattered above and below zero, this suggests that there is no systematic difference between the measurements. Finally, we calculated the sensitivity, specificity, positive predictive value (PPV), negative predictive value (NPV), and the number of false negatives and false positives of the dengue IgG ELISA results using DBS compared with that using sera.

We used Stata 14.0 (StataCorp, College Station, TX, 2015) to compare the participant groups and to calculate the performance indicators. We used Excel (Microsoft, Redmond, WA, 2018) to create the scatterplot with a trend line and to calculate the *R*^2^ value and to generate the Bland–Altman plot.

## RESULTS

**Figure 1.**
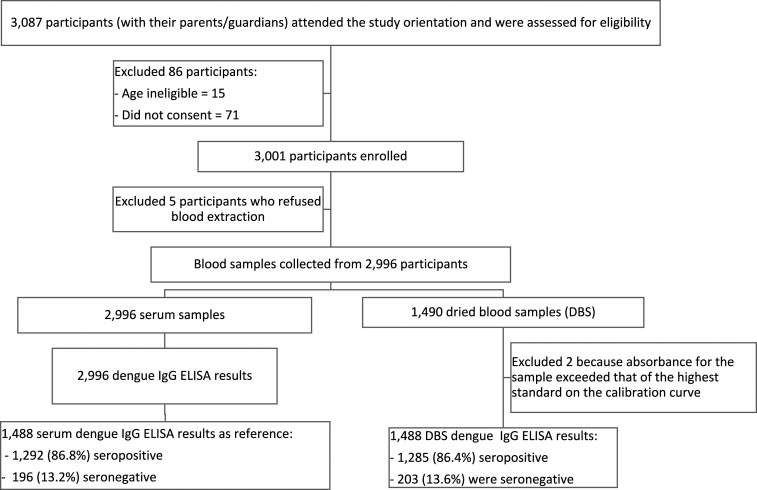
Schema of specimens collected.

From May 2 to June 2, 2017, 3,087 children and their parents or guardians attended the study orientation and were assessed for eligibility (Figure 1). We enrolled 3,001 (97.2%) participants into the study. We collected sera from 2,996 and DBS from the first 1,490 (49.7%) participating children. All 2,996 serum samples and 1,490 DBS were tested by dengue IgG ELISA. Two of the 1,490 (0.1%) DBS were excluded because the absorbance value for the samples exceeded the linear portion of the standard curve of the serially diluted comparator, from which the ELISA index values are determined. We compared the demographic characteristics of the 2,996 participants enrolled in the study with the 1,488 with serum and DBS tested by IgG ELISA (Table 1). The 1,488 children in the subsample were slightly younger with a mean (SD) age of 10.4 (1.3) years than 10.6 (1.5) years in the cohort (*P* < 0.0001). There were no statistically significant differences in gender and study area.

**Table 1:** Comparison* of demographic characteristics of all participants in the cohort with those whose serum and DBS samples were tested by dengue IgG indirect ELISA

		Participants enrolled in the study (*n* = 2,996), *n* (%)	Participants whose serum and DBS samples were tested by dengue IgG indirect ELISA (*n* = 1,488), *n* (%)	*P*-value
Gender	Male	1,446 (48.3)	735 (49.4)	0.476
	Female	1,550 (51.7)	753 (50.6)	
Age (years)	Age, mean (SD)	10.4 (1.3)	10.6 (1.5)	< 0.0001
	Median (minimum, maximum)	10 (8, 14)	10 (8, 14)	
Study area	Bogo	1,557 (52.0)	741 (49.8)	0.171
	Balamban	1,439 (48.0)	747 (50.2)	

DBS = dried blood spots.

* We used the *t*-test for continuous variables (age) and chi-squared test for categorical variables (gender and study area).

**Figure 2.**
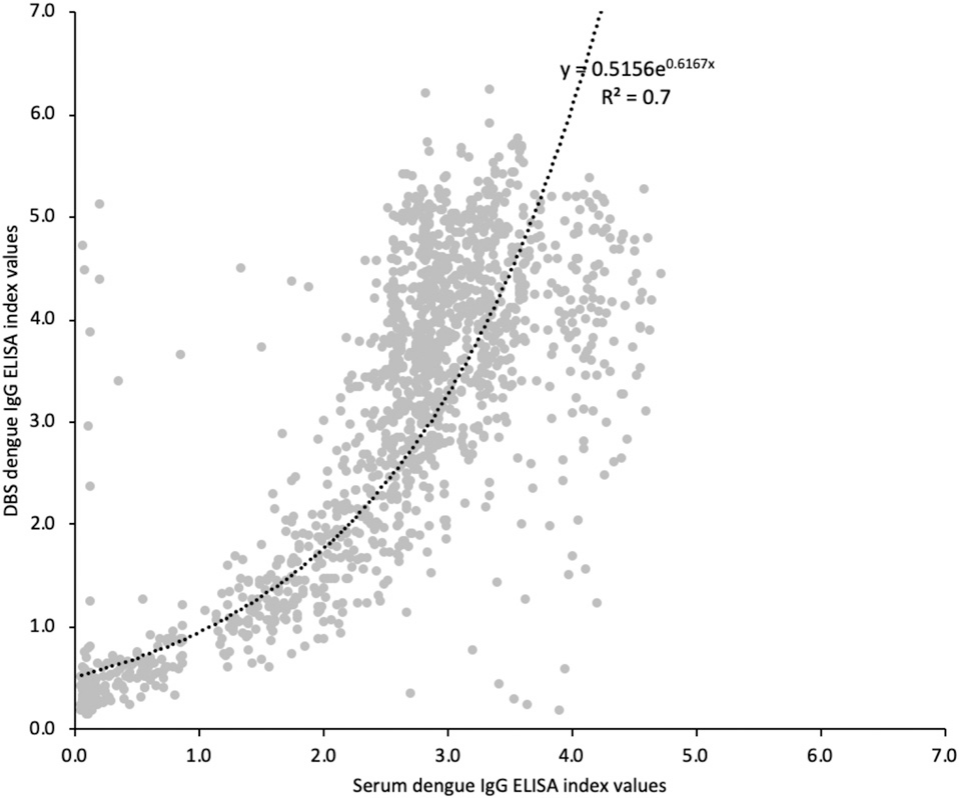
Correlation between dengue IgG indirect ELISA index values using serum samples and dried blood spots (DBS), with an exponential trend line and its equation and the *R*-squared (*R*^2^) value.

**Figure 3.**
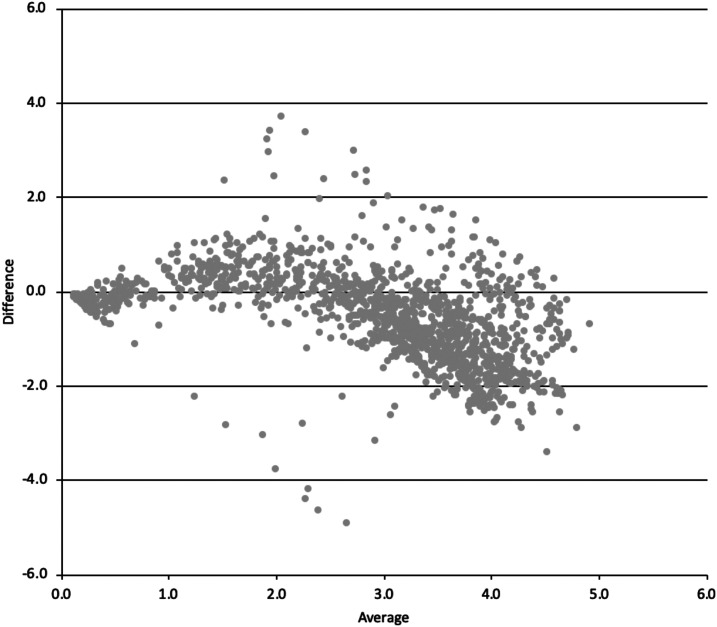
Bland–Altman plot of serum samples and dried blood spots (DBS) dengue IgG indirect ELISA index values, where 1) x-axis (Average) = (serum index value + DBS index value)/2 y-axis (Difference) = serum index value − DBS index value.

We plotted the 1,488 dengue IgG ELISA results using DBS with those using serum samples (Figure 2). We fitted an exponential trend line. The *R*^2^ value was 0.7, indicating a moderate correlation. The Bland–Altman plot is shown in Figure 3. The ELISA results using serum showed a higher signal than DBS in samples with index values between 0.5 and 2. However, for samples with index values greater than 2.5, the DBS results were generally higher than those of serum.

Using an ELISA index value cutoff of 0.9, 1,285/1,488 (86.4%) of the DBS were seropositive and 203 (13.6%) were seronegative, compared with 1,292/1,488 (86.8%) seropositive and 196 (13.2%) seronegative serum samples (Figure 1, Table 2). The DBS method had a sensitivity of 98.3% (95% CI: 97.5–98.9%), specificity of 92.4% (87.7–95.7%), 98.9% (98.2–99.3%) PPV, and 89.2% (84.4–92.6%) NPV compared with sera (Table 2).

**Table 2:** Performance of dengue IgG indirect ELISA using DBS compared with serum samples

		Serum samples		Total
		Seronegative	Seropositive	
DBS	Seronegative	181	22	203
	Seropositive	15	1,270	1,285
	Total	196	1,292	1,488

DBS = dried blood spots. Sensitivity (95% CI): 98.3% (97.5–98.9%). Specificity (95% CI): 92.4% (87.7–95.7%). Positive predicted value (95% CI): 98.9% (98.2–99.3%). Negative predicted value (95% CI): 89.2% (84.4–92.6%). False negative = 22. False positive = 15.

## DISCUSSION

Compared with using serum, the DBS method had a sensitivity of 98.3% and specificity 92.4%. The slight loss in sensitivity may be due to degradation of antibody in the filter paper samples. Alternatively, because the DBS contains whole blood, it is possible that binding of antibodies to red blood cells affects the bioavailable antibody for detection. The larger reduction in specificity could have been from contamination during the punching procedure,^8^ despite the cleaning of the puncher tip with alcohol between samples. To reduce contamination risks, perforated filter paper cards that allow spots to be removed with a pipette tip could be used, but they are not widely available.^8^

We previously reported that based on neutralization testing of 1,961 samples and imputed results of 1035 samples from the 2,996 children, 320 (10.7%) were dengue naive and 2,676 (89.3%) were seropositive for previous dengue virus infection.^7^ In this subset of 1,488, 86.4% and 86.6% were seropositive using DBS and sera, respectively. These high dengue seroprevalence estimates are consistent with previous reports. A baseline assessment before a phase 3 vaccination trial of 604 Filipino children in Cebu and Laguna by plaque-reduction neutralization assay of sera showed dengue seropositivity of 58% in the 2- to 4-year age-group increasing to 93% in those aged 13–16 years.^1^ In another cohort study in Cebu city, dengue hemagglutination inhibition assay of sera showed that the proportion of 1,008 participants having a multi-typic dengue serologic profile increased from 40% in the 6 months to 5-year-old age-group to 99% in those aged 31–50 years.^13^

Several studies in dengue-endemic countries have evaluated DBS or dried serum spot (DSS) samples compared with sera for the detection of anti-DENV IgM and IgG antibodies.^8,14–24^ We tabulated the number and characteristics of participants, serologic assay used, and the sensitivity and specificity of filter paper samples compared with sera in these previous studies (Supplemental Material). Sample sizes ranged from 44 to 781. The previous studies generally tested specimens from patients with suspected dengue infection. Because dengue IgM and IgG antibody levels are higher during ongoing or recent dengue infections, these studies are not quite comparable to our study, which estimates seroprevalence in a healthy population. There was one previous study which estimated dengue incidence using pre- and postseason samples from healthy participants.^20^ In that study, the detection of dengue IgG using 288 DBS specimens compared with serum samples had a sensitivity of 86.0% and specificity of 92.4%.

The impact of dengue on communities could be monitored better if inexpensive and efficient sampling tools for repeated epidemiological surveillance are available. Dengue seroprevalence data could potentially be used to prioritize areas for deployment of population-level dengue control programs, such as the recently reported use of *Wolbachia*-infected mosquitoes to control dengue transmission.^25^ Dried blood spot sampling offers new opportunities for serologic surveillance, especially when repeated measurements are necessary.^24^ Collection of DBS is easy to perform and requires minimal equipment and training.

The time and conditions for storage of filter paper samples for dengue antibody testing have not been standardized.^25^ We stored our DBS in −80°C for 2 years, and others have kept their DBS in −20°C,^17^ in 4°C,^20,21^ in an air-conditioned room^18^ or at room temperature^19^ before testing with good results. Based on a review on the use of filter paper samples in viral diagnostics and epidemiologic studies, room temperature storage should not exceed 2 weeks.^24^

Our study has several limitations. First is the use of the IgG ELISA, which has shortcomings related to specificity and reduced sensitivity for detecting primary infections. Although the assay may not be appropriate for individual testing of dengue serostatus, it is recommended for population-based serosurveys.^5^ Second, we found that the DBS method had a loss of signal at the lower tittered antibody range and an increase in signal at the higher antibody titer. We are uncertain why this pattern occurred, whether this is due to antibodies being bound to red blood cells and eluted better at higher concentrations or some type of cross-reactivity. Third, the blood samples blotted onto each filter paper were collected by venipuncture, not by finger prick. Our results may not reflect the performance of DBS when collected using capillary blood samples. Fourth, the study may have been more informative if the DBS had been kept at ambient temperatures during shipment to replicate a real-world scenario, avoiding the costs and logistics of cold chain shipping. Fifth, our participants belonged to a narrow age-group and had a high seroprevalence of dengue antibodies. Our results may not be generalizable to other populations in other areas with lower dengue seroprevalence. The children in the subsample were slightly younger than those in the whole cohort. The significant difference in age is likely due to the large sample size, with the younger children coming during the earlier part of the survey and being included in the collection of DBS samples. However, we do not think that this is of consequence in this narrow age-group.

In summary, we compared the performance of DBS with serum samples for the detection of dengue IgG by ELISA, using a large number of samples. The DBS method had an acceptable sensitivity and specificity and could be a practical alternative to sera when conducting large population serosurveys to measure DENV seroprevalence.

## Supplemental material

Supplemental materials
