# Supplementary material for: Performance of Dried Blood Spots Compared with Serum Samples for Measuring Dengue Seroprevalence in a Cohort of Children in Cebu, Philippines
Source: Am J Trop Med Hyg. 2020 Nov 2;104(1):130–5. doi: 10.4269/ajtmh.20-0937 (PMC7790110; doi:10.4269/ajtmh.20-0937)
Supplement: Supplementary file 1 [file tpmd200937.SD1.pdf]

Supplementary material

AJTMH-20-0937, The performance of dried blood spots compared with serum samples for measuring dengue seroprevalence in a cohort of children in Cebu, Philippines by Daag, Jedas; Ylade, Michelle; Jadi, Ramesh; Adams, Cameron; Cuachin, Anna; Alpay , Riacarl ; Aportadera, Emma ; Yoon, In-Kyu; de Silva, Aravinda; Lopez, Anna; Deen, Jacqueline

Table S1. Studies evaluating dried serum or blood on filter paper compared with serum samples for detection of anti-DENV antibodies

Reference	Country	Dengue antibody detection	Participants	Serologic assay	Specimens compared*	Sensitivity of DBS/DSS versus sera	Specificity of DBS/DSS versus sera
Kuno, 1991 [1]	Puerto Rico	IgM and IgG	patients with suspected acute dengue infection	In-house enzyme-linked immunosorbent assay (ELISA)	• Sera<br>• DBS	97 IgM<br>96 IgG	97 IgM<br>91 IgG
Ruangturakit, 1994 [2]	Thailand	IgM and IgG	**133** patients with suspected acute dengue infection	In-house ELISA	• Sera<br>• DBS<br>• DSS	N/A	N/A
1996 [3]	Costa Rica, Nicaragua and Cuba	IgM	**118** patients with suspected acute dengue infection	Dengue IgM kit (Institute of Tropical Medicine, Cuba).	• Sera<br>• DBS	98.1%	98.5%
de la C. Herrera, 2006 [4]	Cuba	IgM	**189** patients with suspected acute dengue infection	Dengue IgM Ultramicro ELISA (UMELISA)	• Sera<br>• DBS	92.1%	98.6%
Tran, 2006 [5]	Vietnam	IgM and IgG	**781** febrile patients provided acute and convalescent sera	Dengue IgM capture ELISA (Focus diagnostics, Cypress, CA, USA) and indirect IgG ELISA (Focus diagnostics, Cypress, CA, USA)	• Sera<br>• DBS	N/A	N/A
Matheus, 2008	French	IgM	**130** patients with	In-house ELISA	• Sera	81.6%	74.9%

[6]	Guiana		suspected acute dengue infection (93 samples were collected 1-4 days after the onset of fever and 37 samples after 4th day)		• DBS		
Balmaseda, 2008 [7]	Nicaragua	IgA, IgM and IgG	**356** febrile patients provided acute and convalescent sera<br>**322** participants provided pre- and post-dengue season samples to assess incidence	In-house ELISA	• Sera<br>• DBS	95.6%-IgM for diagnosis<br>59.2%-IgM for incidence<br>86.0%-IgG for incidence	88.9%-IgM for diagnosis<br>81.1%-IgM for incidence<br>92.4%-IgG for incidence
Anders, 2012 [8]	Vietnam	IgM and IgG	**44** patients with NS1 positive acute dengue infection	Capture IgM and IgG (MAC/GAC) ELISA	• Sera<br>• DBS	100%	100%
Chakravarti, 2013 [9]	India	IgM	104 suspected cases of dengue fever	N/A	• Sera<br>• DBS	N/A	N/A
Maldonado-Rodríguez, 2017 [10]	Mexico	IgG (IgM**)	**66** patients with suspected acute dengue infection (blood was collected about 4 days after the onset of fever)	Dengue Virus IgG kit (DRG International, Inc., USA)	• Sera<br>• DSS	94.2%	93.3%

* DBS – dried blood spots; DSS - dried serum spots

** Only four samples were positive for IgM in the serum assay and only one of these was also positive in the DSS assay.

References

1. Kuno G, Gomez I, Gubler DJ, 1991. An ELISA procedure for the diagnosis of dengue infections. J Virol Methods 33: 101-13.
2. Ruangturakit S, Rojanasuphot S, Srijuggravanvong A, Duangchanda S, Nuangplee S, Igarashi A, 1994. Storage stability of dengue IgM and IgG antibodies in whole blood and serum dried on filter paper strips detected by ELISA. Southeast Asian J Trop Med Public Health 25: 560-4.
3. 1996. Utilization of blood samples taken on filter paper for detection of IgM antibodies to dengue virus. Epidemiol Bull 17: 11.
4. de la CHR, Cabrera MV, Garcia S, Gilart M, 2006. IgM antibodies to dengue virus in dried blood on filter paper. Clin Chim Acta 367: 204-6.
5. Tran TN, de Vries PJ, Hoang LP, Phan GT, Le HQ, Tran BQ, Vo CM, Nguyen NV, Kager PA, Nagelkerke N, Groen J, 2006. Enzyme-linked immunoassay for dengue virus IgM and IgG antibodies in serum and filter paper blood. BMC Infect Dis 6: 13.
6. Matheus S, Meynard JB, Lacoste V, Morvan J, Deparis X, 2007. Use of capillary blood samples as a new approach for diagnosis of Dengue virus infection. J Clin Microbiol 45: 887-90.
7. Balmaseda A, Saborio S, Tellez Y, Mercado JC, Perez L, Hammond SN, Rocha C, Kuan G, Harris E, 2008. Evaluation of immunological markers in serum, filter-paper blood spots, and saliva for dengue diagnosis and epidemiological studies. J Clin Virol 43: 287-91.
8. Anders KL, Nguyet NM, Quyen NTH, Ngoc TV, Tram TV, Gan TT, Tung NT, Dung NT, Chau NVV, Wills B, Simmons CP, 2012. An evaluation of dried blood spots and oral swabs as alternative specimens for the diagnosis of dengue and screening for past dengue virus exposure. Am J Trop Med Hyg 87: 165-170.
9. Chakravarti A, Siddiqui O, Malik S, Uppal B, 2013. Use of dried blood blotted on filter paper to detect dengue IgM antibody and dengue NS1 antigen. Southeast Asian J Trop Med Public Health 44: 226-31.
10. Maldonado-Rodriguez A, Rojas-Montes O, Vazquez-Rosales G, Chavez-Negrete A, Rojas-Uribe M, Posadas-Mondragon A, Aguilar-Faisal L, Cevallos AM, Xoconostle-Cazares B, Lira R, 2017. Serum Dried Samples to Detect Dengue Antibodies: A Field Study. Biomed Res Int 2017: 7215259.
